# A selective Cu^II^ complex with 4-fluorophenoxyacetic acid hydrazide and phenanthroline displays DNA-cleaving and pro-apoptotic properties in cancer cells

**DOI:** 10.1038/s41598-021-03909-1

**Published:** 2021-12-27

**Authors:** Pedro Henrique Alves Machado, Drielly Aparecida Paixão, Ricardo Campos Lino, Tiago Rodrigues de Souza, Nayara Júnia de Souza Bontempo, Luana Munique Sousa, Fernanda Van Petten de Vasconcelos Azevedo, Priscila Capelari Orsolin, Paula Marynella Alves Pereira Lima, Isabella Castro Martins, Joyce Ferreira da Costa Guerra, Samuel Cota Teixeira, Thaise Gonçalves Araújo, Luiz Ricardo Goulart, Sandra Morelli, Wendell Guerra, Robson José de Oliveira Júnior

**Affiliations:** 1 Instituto de Biotecnologia, Universidade Federal de Uberlândia, Uberlândia, MG Brazil; 2 Instituto de Química, Universidade Federal de Uberlândia, Uberlândia, MG Brazil; 3 Instituto de Ciências Biomédicas, Universidade Federal de Uberlândia , Uberlândia, MG Brazil; 4 Centro Universitário de Patos de Minas , Patos de Minas, MG Brazil

**Keywords:** Cancer therapy, Cancer, Drug discovery, Cell death, Chemical synthesis

## Abstract

The thin line between efficacy and toxicity has challenged cancer therapy. As copper is an essential micronutrient and is important to tumor biology, Cu^II^ complexes emerged as an alternative to chemotherapy; however, its biological properties need to be better understood. Thus, we report in vitro the antitumor effects of two Cu^II^ complexes named [Cu(4-fh)(phen)(ClO_4_)_2_] (complex **1**) and [Cu(4-nh)(phen)(ClO_4_)_2_]·H_2_O (complex **2**), in which 4-fh = 4-fluorophenoxyacetic acid hydrazide; 4-nh = 4-nitrobenzoic hydrazide and phen = 1,10-phenanthroline. Both complexes presented cytotoxic activity against tumor cells, but only complex **1** showed significant selectivity. Complex **1** also induced DNA-damage, led to G0/G1 arrest and triggered apoptosis, which was initiated by an autophagy dysfunction. The significant in vitro selectivity and the action mechanism of complex **1** are noteworthy and reveal this prodrug as promising for anticancer therapy.

## Introduction

The understanding of pharmacological properties of different substances, associated with the evolution of laboratory techniques related to the bioinorganic profile, allowed the industrial-scale synthesis of molecules with anti-inflammatory, anticancer and antibacterial properties^1^. In this context, previous studies have shown that metal complexes bearing hydrazides and derivatives are generally more effective than their respective free ligands^2–7^. For example, a series of Pt^II^–hydrazide complexes present higher inhibitory effect towards Friend leukemia cells in comparison with their respective free ligands and cisplatin^8,9^. Pt^II^ complexes bearing cyclopentanecarboxylic acid hydrazide induce apoptosis and exert significantly lower in vivo toxicity as compared with cisplatin^10^. In fact, metal complexes bearing hydrazides have a great pharmacological potential to be explored and the chelation of these ligands with metal ions plays an important role in the improvement of biological properties^7^.

Copper is an essential trace element for the function of enzymes involved in DNA synthesis, energy metabolism and respiration in all living organisms^11^. These properties have been taken into account for the design of new copper complexes with antitumor action, since endogenous metals may be less toxic^1,12^. Special attention has been devoted to Casiopeinas, which have entered phase I clinical trials in Mexico^13,14^. Among them, Cas III-ia induces cell death by autophagy and apoptosis, through the activation of reactive oxygen species (ROS) dependent on c-Jun NH2-terminal kinase (JNK) signaling^15^. Indeed, there is a great interest in the use of copper complexes in cancer chemotherapy and those containing *N,N*-heterocyclic ligands are noteworthy^16^.

Despite the exceptional biological activity demonstrated by copper complexes and hydrazides, the literature reports few studies on the anticancer activity of copper compounds containing these ligands. Four Cu^II^ complexes with general formula [Cu(N–O)(N–N)(acetonitrile)(ClO_4_)]ClO_4_, where N–O = 2-furoic acid hydrazide or 2-thiophenecarboxylic acid hydrazide and N–N = 2,2-bipyridine or 1,10-phenanthroline, were previously described and inhibited cellular growth in a concentration-dependent manner, with higher cytotoxicity in comparison to free ligands^17^. Moreover, under UV-light exposure, they enhanced the DNA cleavage activity^18^.

Our research group also reported the antitumor effect of ternary Cu^II^ complexes [Cu(N–O)(N–N)(ClO_4_)_2_], in which N–O = 4-fluorophenoxyacetic acid hydrazide (4-fh) or 4-nitrobenzoic hydrazide (4-nh) and N–N = 1,10-phenanthroline (phen), 4–4′-dimethoxy-2–2′-bipyridine (dmb) or 2,2-bipyridine (bipy). Among these compounds, those containing fluorophenoxyacetic acid hydrazide or 4-nitrobenzoic hydrazide and 1,10-phenanthroline, herein called complex **1** and complex **2**, respectively, were more effective against three tumor cell lines inducing apoptosis in MDA-MB-231 cell line^16^. We have also verified that Cu^II^ complexes with phenanthroline were more active against tumor cell lines than their bipyridine analogues, since the planar polycyclic phenanthroline ring interacts better with DNA^19^. Considering that [Cu(4-fh)(phen)(ClO_4_)_2_] **1** and [Cu(4-nh)(phen)(ClO_4_)_2_]·H_2_O **2** are promising candidates as anticancer agents, we herein aimed to assess their biological activities in tumor cells. In fact, cancer is still a public health problem being the second leading cause of death in the world, totaling 9.6 million deaths in 2018. Globally, one in six deaths is related to this disease^20^. Therefore, the need for new drugs for the treatment of this non communicable disease is evident, with higher effectiveness and lower toxicity^21^.

## Results

### Cytotoxicity studies

**Figure 1:**
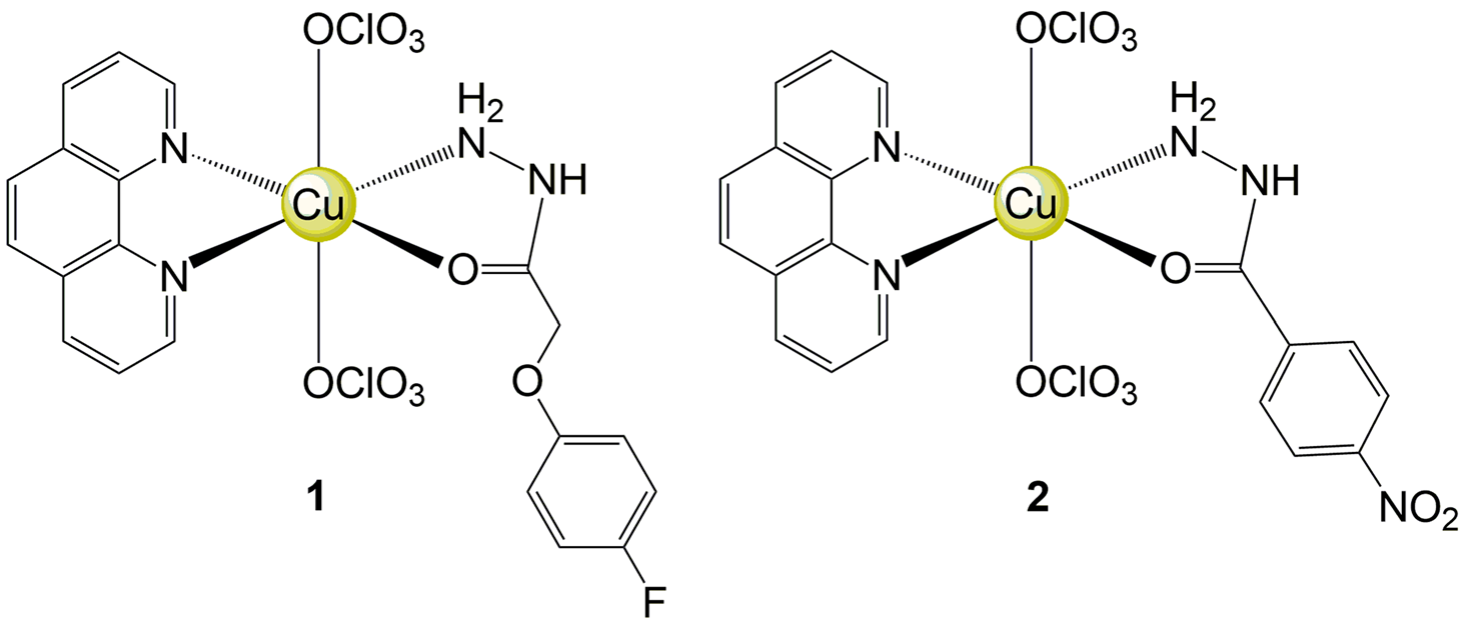
Chemical structure of two Cu^II^ complexes named [Cu(4-fh)(phen)(ClO_4_)_2_]-complex **1**, and [Cu(4-nh)(phen)(ClO_4_)_2_]·H_2_O-complex **2** (4-fh = 4-fluorophenoxyacetic acid hydrazide, 4-nh = 4-nitrobenzoic hydrazide and phen = 1,10-phenanthroline).

Two complexes (Fig. 1) were prepared according to the procedure previously published by our group^16^. The X-ray structural analysis and EPR data confirmed that the geometry around the Cu^II^ ion is octahedral distorted, with two perchlorate anions occupying the apical positions. Nevertheless, conductivity measurements revealed that complexes of the type [Cu(N–O)(N–N)]^2+^ are produced in a solution. We also verified the stability of these species, an important factor for the development of clinical metal-based drugs, and they were stable in a mixture containing H_2_O/DMSO 0.1% with or without buffer for at least 6h^16^. In previous in vitro assays performed by our group, it was found that these complexes (**1** and **2**) displayed high cytotoxic activity, being more active than carboplatin, free ligands and [Cu(phen)_2_]^2+^ towards K562, MDA-MB-231 and MCF-7 cell lines. These studies also indicated that **1** and **2** induce apoptotic cell death in MDA-MB-231 cell line and bind to DNA with K values of 4.38 × 10^4^ and 2.62 × 10^4^, respectively^16^. Considering these aspects, the evaluation of these compounds regarding selectivity is highly desirable, as well as the understanding of their mechanisms of action in detail.

As demonstrated in Table 1, the resazurin reduction assay demonstrated that both complexes were active against the Sarcoma 180 cell line displaying IC_50_ values comparable to cisplatin. It is interesting to note that complex **1** was slightly more cytotoxic than complex **2**, with IC_50_ value of 4.16 µM. Although complex **2** also presented high cytotoxicity against Sarcoma 180 cell line (IC_50 Sarcoma 180_ = 5.15 µM), it was not selective (SI < 2). Thus, complex **1** was selected for further studies because it showed excellent selectivity index values against Sarcoma 180 cell line (IC_50_ = 4.16 µM and SI = 4.77) and expressed high activity and selectivity against the BF16F10 melanoma cell line (IC_50_ = 1.83 µM and SI > 10). The IC_50_ observed in the non-tumoral myocyte C2C12 cell line was much higher than the one observed in tumoral cell lines (IC_50_ = 19.85).

**Table 1:** IC_50_ values for the complexes **1** and **2** on tumor (Sarcoma 180 and melanoma B16-F10) and non-tumor (myoblast C2C12) cell lines.

Complex	IC_50_^a^ (µM)			SI	
	Sarcoma 180	B16F10	C2C12		
**1**	4.16 ± 2.3	1.83 ± 0.05	19.85 ± 4.7	4.77^b^	10.85^c^
**2**	5.15 ± 2.8	n.d	4.06 ± 2.5	0.79^b^	n.d^c^
Cisplatin	2.44 ± 0.47	n.d	18.31 ± 1.95	7.50^b^	n.d^c^

^a^Concentration required to inhibit 50% of cell growth ± SD.

^b^SI (Selectivity Index) = IC_50 C2C12_/IC_50 SARCOMA 180_.

^c^SI (Selectivity Index) = IC_50 C2C12_/IC_50 B16F10_.

n.d. value not determined.

**Figure 2:**
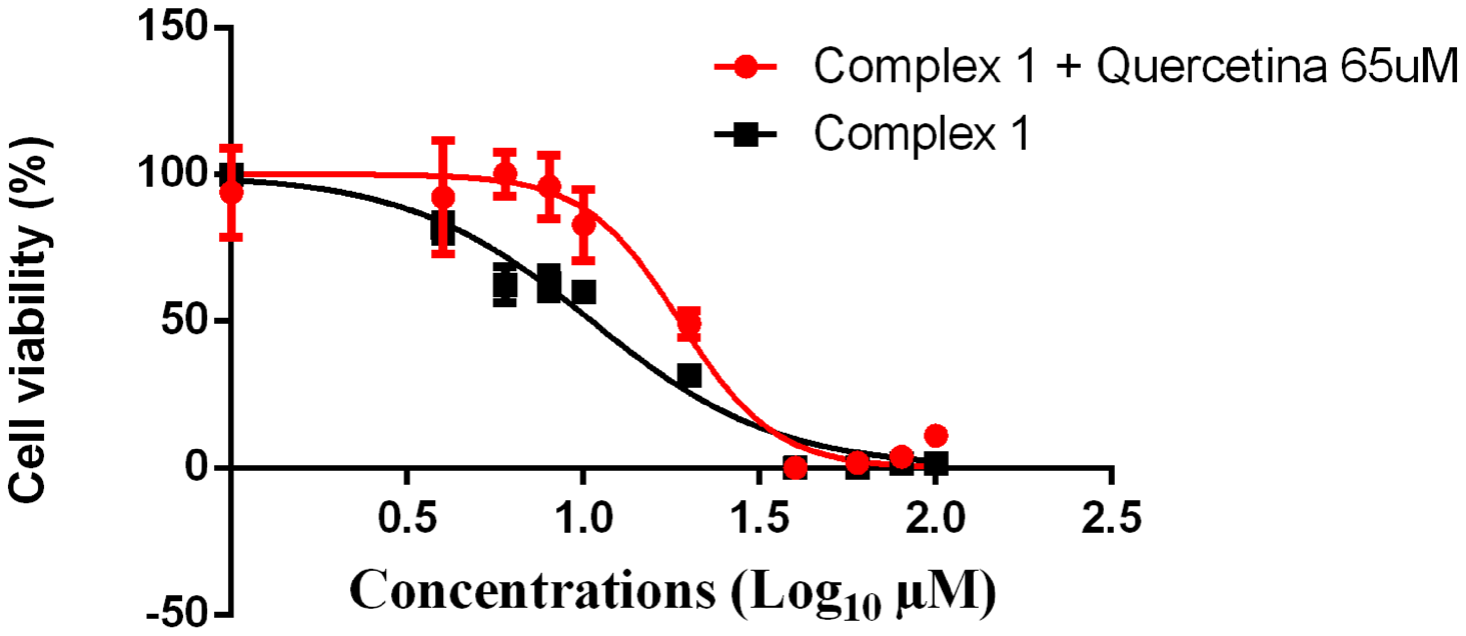
Sigmoidal dose–response curves of sarcoma 180 treatment with complex **1** alone (black points) and complex **1** associated with quercetin (red points) for 24 h in the resazurin reduction assay. Each point represents the means ± standard deviation for n = 4 replicates.

**Figure 3:**
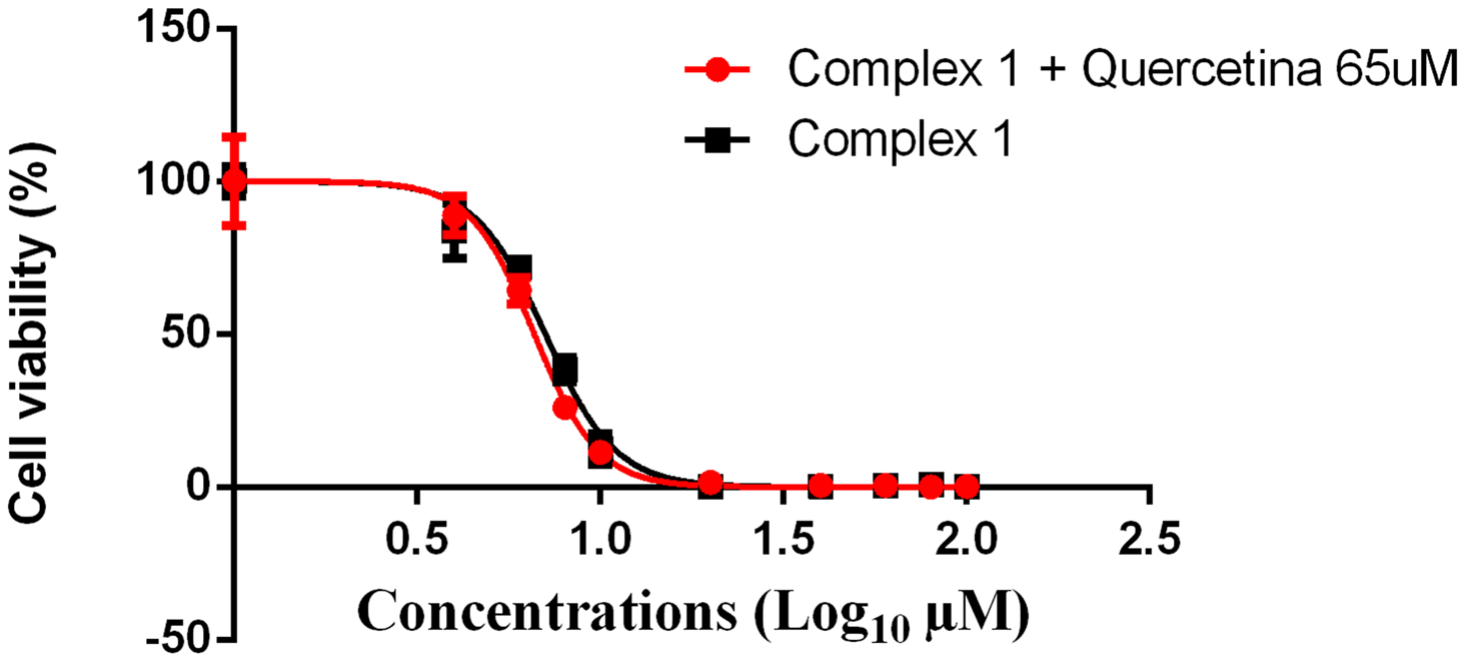
Sigmoidal dose–response curves of melanoma B16F10 treatment with complex **1** alone (black points) and complex **1** associated with quercetin (red points) for 24 h in the resazurin reduction assay. Each point represents the means ± standard deviation for n = 4 replicates.

Cell death induced by copper complexes has been previously associated with increased levels of ROS. Hence, we used quercetin, a known ROS scavenger, to verify the effects of complex **1** in the oxidative pathway. Sarcoma 180 and melanoma B16F10 were first treated with quercetin for 24 hours, which was cytotoxic only for melanoma B16F10 (IC50 = 110.4 µM) (see Supplementary Fig. S1 online). Subsequently, both cells were treated with complex **1** alone and associated with quercetin for 24 h. The IC_50_ for sarcoma 180 cells increased to 19.82 µM (Fig. 2), when these cells were treated with quercetin, indicating the ability of quercetin to attenuate cytotoxicity induced by complex **1**, probably due to ROS inactivation. This behavior was not observed for melanoma B16F10 cell line (Fig. 3), whose IC_50_ did not increase significantly when these cells were treated with quercetin.

### Oxidative stress studies

#### ROS generation

**Figure 4:**
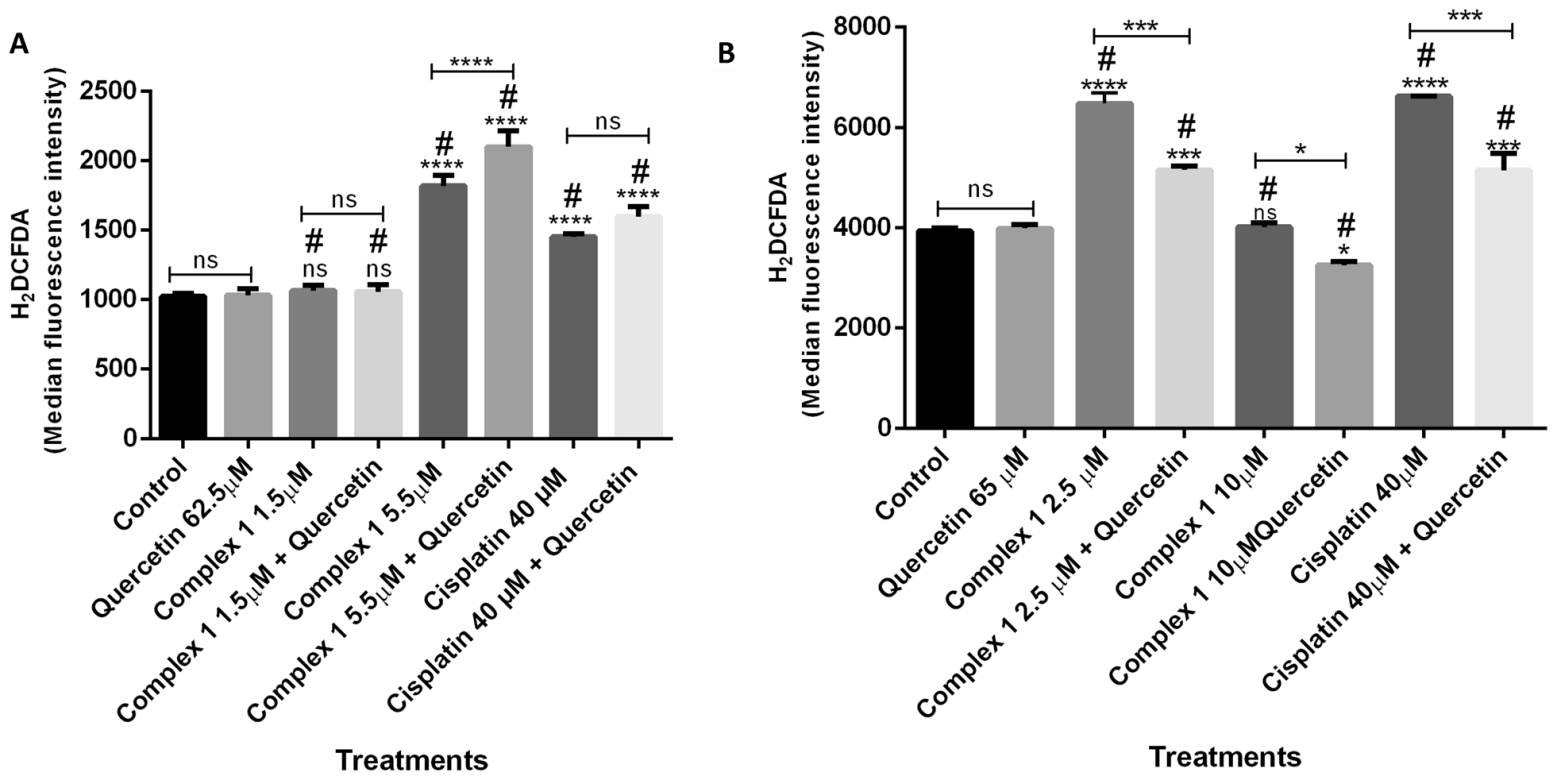
Flow cytometry analyses of the production of reactive oxygen species (ROS) through the quantification of H2DCFDA median fluorescence intensity. Cells were treated with complex **1** and quercetin 62.5 µM. (**A**) Melanoma B16F10 cells treated with 1.5 μM and 5.5 μM of complex **1** (**B**) Sarcoma 180 cells treated with 2.5 μM and 10 μM of complex **1**. Data are presented as means ± the standard deviation. *p < 0.05. **p < 0.01, ***p < 0.001 and ****p < 0.0001. The comparison with the negative control was performed by one-way ANOVA followed by Dunnett’s post-test and the concentrations with or without quercetin were compared to each other by one-way ANOVA followed by Tukey´s post-test.

Sarcoma 180 and melanoma B16F10 cell lines were treated with complex **1** at different concentrations, being 2.5 µM and 10 µM for sarcoma 180, and 1.5 µM and 5.5 µM for melanoma B16F10. Treatments were performed with complex **1** alone or associated with quercetin at 62.5 µM (Fig. 4). In sarcoma 180, complex **1** at 2.5 µM significantly increased ROS levels and quercetin reversed this effect (Fig. 4A). In melanoma B16F10, complex **1** at 5.5 µM significantly increased ROS levels, but the antioxidant quercetin did not reverse this effect (Fig. 4B). Actually, quercetin induced the ROS production in melanoma B16F10 when treated with 5.5 µM of complex **1** and 62.5 µM of quercetin. In both cells, cisplatin induced the ROS production, but quercetin only reversed this effect in sarcoma 180 cells.

#### Lipid peroxidation

**Figure 5:**
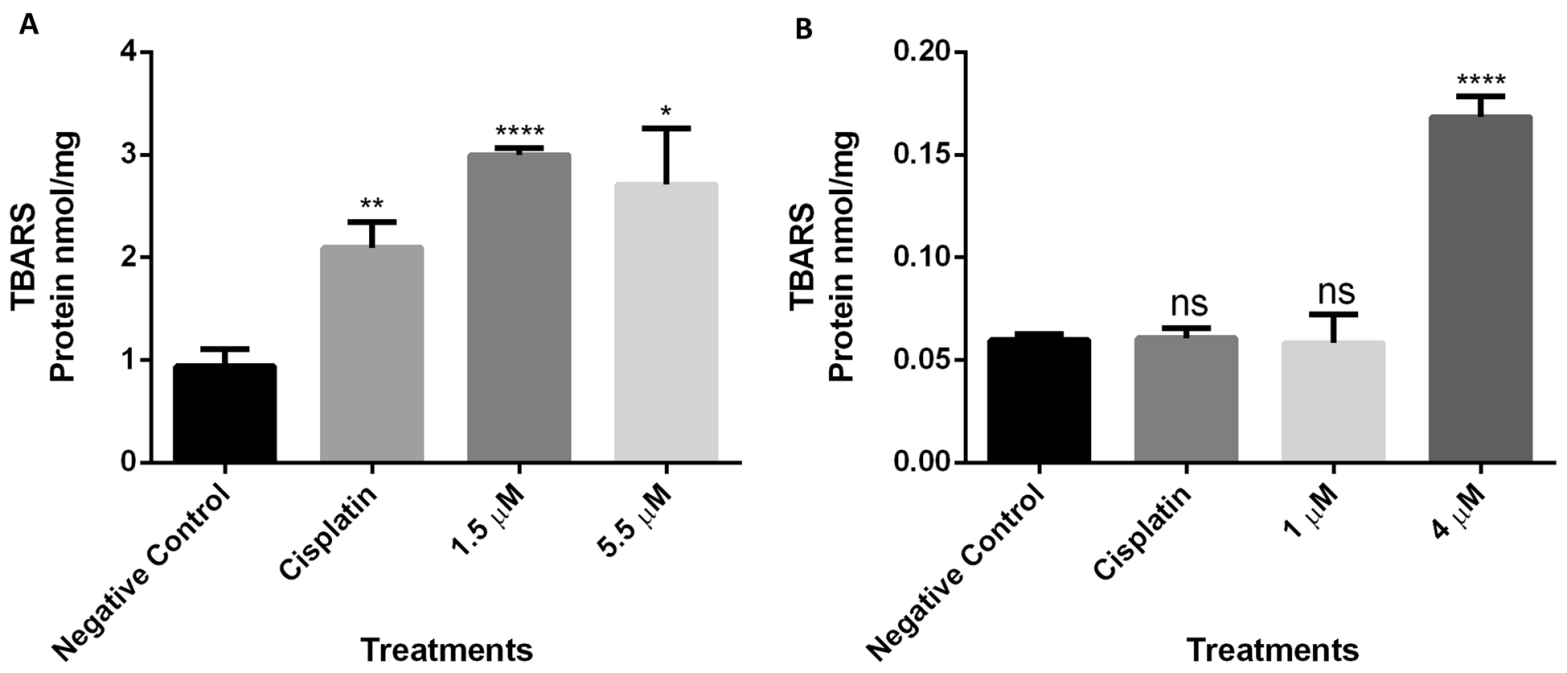
Thiobarbituric Acid Reactive Substances (TBARS) levels of (**A**) melanoma B16F10 and (**B**) sarcoma 180 cell line treated with different concentrations of complex **1**. Data represent the means ± the standard error. *p < 0.05; **p < 0.01 and ****p < 0.0001 (each group compared with the negative control by T-test).

In order to measure the extent of lipid peroxidation induced by complex **1,** the levels of Thiobarbituric Acid Reactive Substances (TBARS) were measured in sarcoma 180 cells treated with concentrations of 1 µM and 4 µM and melanoma B16F10 cells treated with 1.5 µM and 5.5 µM. As shown in Fig. 5, comparing to the negative control, significantly increased lipid peroxidation was detected in melanoma B16F10 cells treated with 1.5 µM and 5.5 µM of complex **1** and in sarcoma 180 cells treated with 4 µM.

### DNA cleavage experiments

#### Genomic DNA degradation of tumor cells

In order to verify the action of complex **1** in the genomic DNA of tumor cells, Sarcoma 180 cells were treated for 24 h with complex **1** at 1 µM, 4 µM and 20 µM. The results showed that complex **1** fragmented the genomic DNA of Sarcoma 180 tumor cells at a concentration of 20 µM (see Supplementary Fig. S2 online).

#### Plasmid DNA cleavage assay

**Figure 6:**
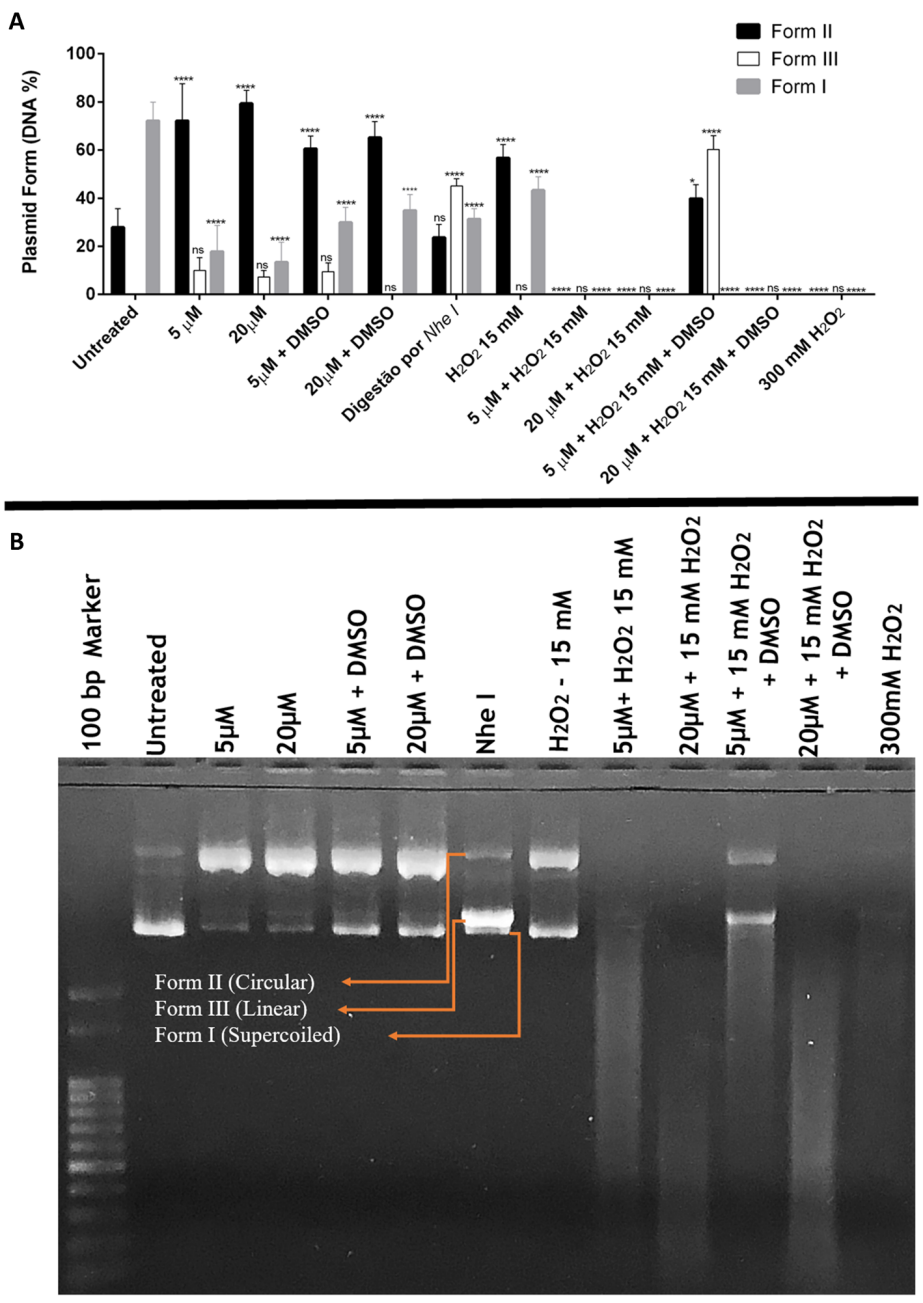
Plasmid siSTRIKE degradation assay (**A**) Quantification of DNA percentage according to the electrophoresis pattern. Data are expressed as means ± standard deviation of three assays. Statistical analysis was performed with 2-way ANOVA and multiple comparison by Dunnett's test. *p < 0.05; **p < 0.01; ***p < 0.001; ****p < 0.0001; ns not significant, compared to untreated control. (**B**) Representative agarose gel electrophoresis of plasmid DNA treated with complex **1**, DMSO, H_2_O_2_ and *Nhe* I restriction enzyme. Circular, linear and supercoiled plasmid forms are indicated in the gel. Full-length gels are presented in Supplementary Figure S5.

In order to evaluate the plasmid cleavage capacity of complex **1**, siSTRIKE plasmid (30 ng) was treated with different concentrations of the compound, in the presence or absence of H_2_O_2_ and DMSO. Complex **1** interacted with plasmid DNA causing breaks (Fig. 6). Gel electrophoresis detected the presence of three band patterns corresponding to the plasmid DNA conformations (Fig. 6B). Form I (supercoiled) refers to the most compact form of the plasmid, without single breaks in the DNA strands, which makes it easier to migrate through the gel network. Form II is the structure regarding breaks in one DNA strand, which makes the conformation more circular or relaxed, being identified as the most superior band of the gel. Form III would be the result of double-strand breaks at DNA molecule, which turns it into the linearized conformation, acquiring an intermediary position in the gel.

When plasmid was treated only with complex **1** the intensity of the band corresponding to plasmid form II (circular) increased at all concentrations of the compound when compared to the negative control (no treatment) (Fig. 6B). When complex **1** was associated with DMSO, which is a free radical scavenger, the band corresponding to form I (supercoiled—intact plasmid) increased, demonstrating that DMSO attenuates the cleavage capacity of the complex.

The restriction enzyme *Nhe*I was used as a positive control for DNA double-strand breaks, since the plasmid contains a restriction site for this enzyme. An intermediary band was generated after restriction, corresponding to form III (linear) (Fig. 6B). It was also possible to notice the appearance of small bands referring to form III in treatments that involved the isolated complex **1** (5 and 20 µM) and in the lowest concentration of the complex **1** (5 µM) associated with DMSO, which indicates that the complex **1** is also capable of inducing double breaks on the DNA strands. The treatment with only H_2_O_2_ (15 mM) simulated an oxidative environment and was responsible for the increase in plasmid form II (circular) (Fig. 6B). However, by associating complex **1** with H_2_O_2_, a total plasmid DNA degradation was observed at the concentrations 5 µM and 20 µM of complex **1** (Fig. 6B). Finally, when this set was associated with DMSO, the lowest concentration of the complex (5 µM) had its action attenuated, since plasmid forms II and III were observed. Thus, the use of DMSO protects DNA from complex **1** damage.

In addition, it is noticeable that the complex **1** causes greater damage to the DNA when in the presence of reactive oxygen species, since the total degradation of plasmid DNA occurred in treatments that included hydrogen peroxide associated with the complex **1** and this situation was reversed when the lowest concentration of the complex was associated with DMSO, reappearing plasmid forms III and II.

### Cell cycle arrest and apoptosis activation analysis

**Figure 7:**
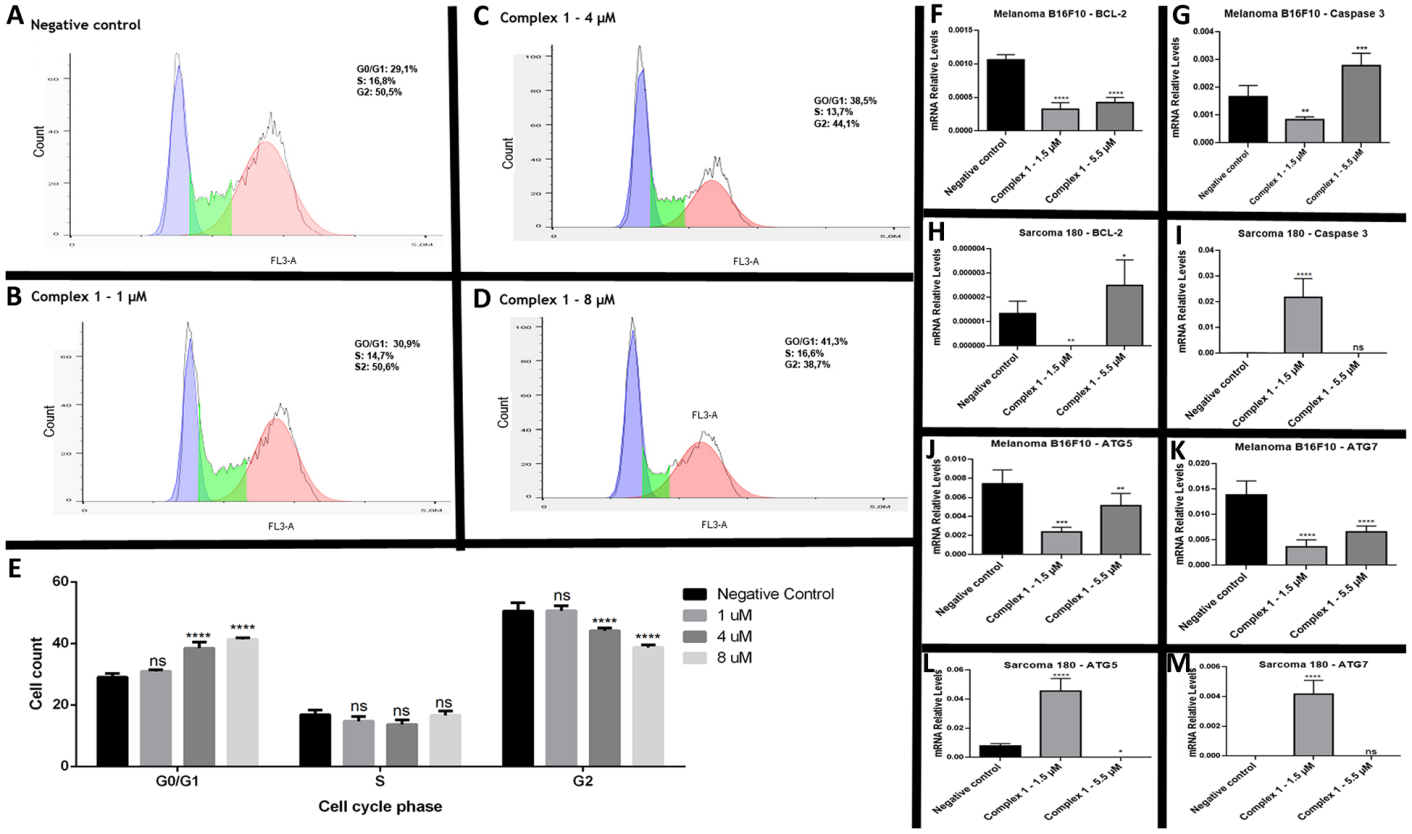
Flow cytometry analyses of the cell cycle distribution and gene expression by qPCR of Sarcoma 180 cells treated with complex **1**. (**A**) Negative control; (**B**) 1 μM; (**C**) 4 μm; (**D**) 8 μM. (**E**) Representative graphic of quantitative analysis of flow cytometry data according to cell cycle phase for the groups indicated. Data represent the means ± the standard error. *p < 0.05. **p < 0.01, ***p < 0.001 and ****p < 0.0001 (compared with the negative control by ANOVA one-way followed by Bonferroni´s post-test). (**F**) mRNA Relative Levels of *BCL-2* gene in melanoma B16F10; (**G**) mRNA Relative Levels of *CSP3* gene of melanoma B16 F10; (**H**) mRNA Relative Levels of *BCL-2* gene of sarcoma 180; (**I**) mRNA Relative Levels of *CSP3* gene of sarcoma 180; (**J**) mRNA Relative Levels of ATG5 gene of melanoma B16F10; (**K**) mRNA Relative Levels of ATG7 gene of melanoma B16F10; (**L**) mRNA Relative Levels of ATG5 gene OF sarcoma 180; (**M**) mRNA Relative Levels of of ATG7 gene of sarcoma 180. Data represent the means ± the standard error. *p < 0.05. **p < 0.01, ***p < 0.001 and ****p < 0.0001 (compared with the negative control by ANOVA one-way followed by Dunnett´s post-test).

In order to assess the effect of complex **1** in the cell cycle, Sarcoma 180 cells were treated with three different concentrations of complex (1 µM, 4 µM and 8 µM) and cell cycle phases were analyzed with flow cytometry. Sarcoma 180 cell cycle was trapped in G0/G1 phase, demonstrating an antiproliferative potential of the complex **1** (Fig. 7A–E). Cell cycle arrest is a response to DNA damage events and may trigger apoptosis. In this context, we treated melanoma B16F10 and sarcoma 180 with complex **1** and performed qPCR to quantify mRNA relative levels of two genes involved in the apoptosis pathway, B-cell lymphoma 2 (BCL-2) and caspase 3 (CSP3), and two genes of the autophagic pathway (ATG5 and ATG7), once autophagy can also trigger apoptosis.

After treatment with complex **1,** *BCL-2* transcript levels were downregulated in melanoma B16F10 (Fig. 7F) in both tested concentrations. *CSP3* expression was upregulated (Fig. 7G) in the higher concentration, indicating that the apoptotic cascade was activated. In Sarcoma 180, *BCL-2* mRNA levels were upregulated at the lower concentration of complex **1** (Fig. 7H) and higher levels of *CSP3* were identified when cells were treated with a higher concentration of complex **1** (Fig. 7I).

In light of the fact that the treatment with complex **1** generated ROS, induced genomic and plasmid DNA degradation and arrested cells in G0/G1, we investigated whether complex **1** could be effective in a human tumor cell. In this way, we evaluated by which mechanism cell death of HeLa tumor cells was induced when treated with complex **1**. All tested concentrations of the complex **1** (1 µM, 4 µM and 10 µM) induced apoptosis of HeLa tumor cells (see Supplementary Fig. S3 online). In addition, we performed a time lapse of HeLa cells treated with 100 µM of the complex **1** and were able to observe alterations in cell morphology (formation of blebbing) related to the apoptotic process (see Supplementary Fig. S3 online).

### Autophagy activation analysis

**Figure 8:**
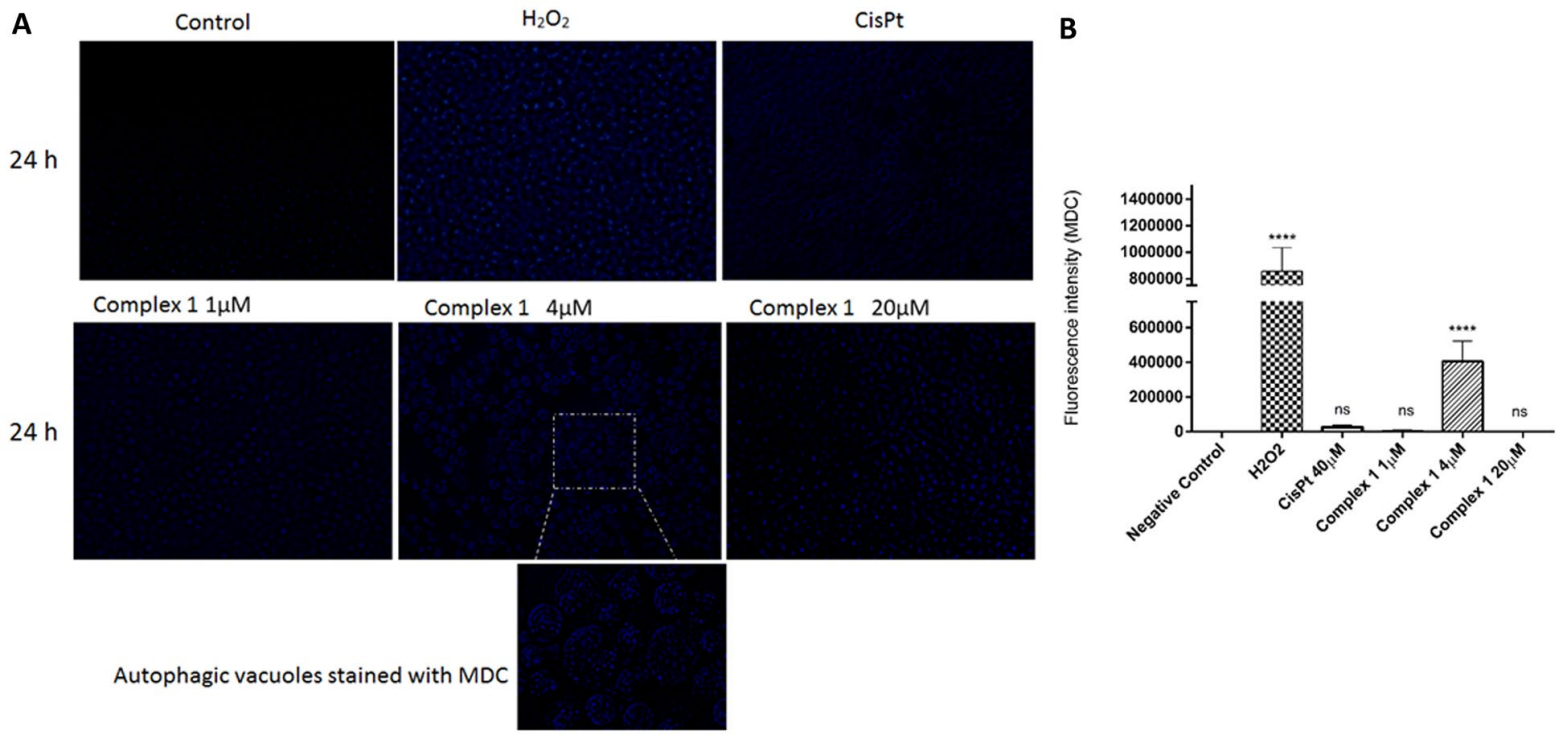
(**A**) Monodansylcadaverine staining of autophagic vacuoles of Sarcoma 180 cell line treated with complex **1** (1 μM, 4 μM and 20 μM). (**B**) Quantitative analyses of the fluorescence intensity of monodansylcadaverine after treatments. Data represent the means ± the standard error. *p < 0.05. **p < 0.01, ***p < 0.001 and ****p < 0.0001 (compared with the negative control by ANOVA one-way followed by Bonferroni´s post-test).

The analyses of autophagy genes by means of real-time PCR showed that the treatment with complex **1** induced the downregulation of ATG5 (Fig. 7J) and ATG7 (Fig. 7K) genes in melanoma B16F10 in both concentrations tested and in sarcoma 180 induced the upregulation of ATG5 (Fig. 7L) and ATG7 (Fig. 7M) in the lower concentration of complex **1**. Since the overexpression of the autophagic genes ATG5 and ATG7 in sarcoma 180 cells was induced, we performed monodansylcadaverine staining to verify whether the formation of autophagic vacuoles was induced by the treatment with complex **1** at 1 μM, 4 μM and 20 μM. The Complex **1** at the concentration of 4 µM truly activated autophagic pathway in sarcoma 180 tumor cells, once autophagic vacuoles were detected through monodansylcadaverine staining (Fig. 8A,B).

## Discussion

Both complexes synthesized showed cytotoxic properties, but in preliminary tests, complex **1** demonstrated better results such as a high selectivity index, in addition to interesting antitumor properties, making it a candidate for antitumor drugs. The ligands phen and 4-fh or 4-nh did not present significant activity against diverse tumorigenic and non-tumorigenic cells^16,22^. Thus, the chelation to copper ion provided the cytotoxic activity of these compounds.

The Complex **1** demonstrated high rates of selectivity for the Sarcoma 180 and B16-10 tumor cell lines (respectively 4.78 and 10.85), once selective indices are considered as significant when ≥ 3^23,24^, which makes it particularly interesting considering the need for less toxic drugs for tumor treatment. Therefore, complex **1** was selected for further studies. Additionally, when compared to other Cu^II^ complexes containing analogous ligands, complex **1** has already been shown to stand out as one of the most active copper-based compounds^25,26^ with high selectivity^22^, as it can be confirmed by the present work.

Copper complexes can also generate ROS, inducing DNA damage, inhibiting proteasome function^27^, and triggering apoptosis or other cell death pathways^22,28–32^. For these reasons, those mechanisms were further analyzed in order us to describe the biological activities of complex **1.** Regarding the potential of complex **1** in generating ROS, we performed cytotoxicity tests using quercetin, a known antioxidant molecule which has already been described as playing a protective role against copper(II)-induced oxidative stress^33^. We observed that the co-treatment of complex **1** with quercetin increased the IC_50_ of sarcoma 180 cells, demonstration that the ROS generation plays an important role in the complex **1** effect in these tumor cells. However, quercetin did not protect melanoma B16F10 cells from the cytotoxicity effect of our compound; quite the opposite, quercetin significantly enhanced the cytotoxicity of complex **1** in melanoma B16F10. This phenomenon can be explained by the already described melanocytotoxicity exhibited by quercetin, which enhances melanogenesis in melanocytes by increasing the activity and synthesis of tyrosinase. We confirmed this behavior in a cytotoxicity assay with quercetin in both cell lines, in which only melanoma B16F10 cells presented cytotoxicity in the tested concentrations (ranging from 2000 to 3.9 µM) showing an IC_50_ of 110.4 µM^34–36^.

The Complex **1** exhibited the ability to induce the elevation of intracellular ROS in Sarcoma 180 and melanoma B16F10 cell lines. ROS plays important roles within the cell regulation, cell cycle and cell death^22,37–39^. When intracellular levels of ROS are imbalanced, serious damages to biomolecules can be caused^40^, such as proteins, lipids, nucleic acids and whole organelles^41,42^. Many chemotherapeutic drugs increase intracellular generation of ROS, which brings antiproliferative effect^43^ and induces apoptosis^37^. Previous studies have shown that Casiopeinas, which are already undergoing clinical tests, also act through oxidative mechanisms causing related cell damage and death^44^. These biological activities were also observed for the complex **1**. Copper complexes are widely described as ROS inductor agents^22,29,31,43,45^, thus activating autophagic pathway, culminating into apoptosis^22,31,45^.

Lipid peroxidation is a key indicator of oxidative stress. Our results indicate that the increased ROS production induced by complex **1** results in oxidative damage and disruptions in redox homeostasis in the cell. The involvement of lipid peroxidation in the cytotoxicity of copper complexes has been reported^46^. Excessive oxidation of lipids alters the physical properties of cellular membranes and other intracellular structures. Moreover, lipid peroxides are also able to propagate further generation of ROS, or degrade into reactive compounds that can cause covalent modification of proteins and DNA^47^, leading to cell death through apoptosis and autophagy mechanisms^48^.

The DNA is responsible for regulating several cellular functions and if this molecule gets damaged, cell death pathways are triggered^27^. In the literature, DNA fragmentation has been reported for many copper(II) complexes containing 1,10-phenanthroline^22,39,40^. These studies are based on the discovery that [Cu(phen)_2_]^+^ is able to bind/intercalate, and promote DNA cleavage^16,49^. This ability observed for several copper complexes bearing 1,10-phenanthroline may explain the results for complex **1** in the fragmentation of the genomic DNA of Sarcoma 180 tumor cells and in the plasmid DNA cleavage activity.

The data herein obtained demonstrate that complex **1** is effective in damaging plasmid DNA, but with greater activity in an environment rich in free radicals. Thus, the complex is likely to interact with the DNA and cause breaks through ROS induction, via redox mechanisms, corroborating with several other authors^22,50–57^.

Moreover, copper complex associated with 1,10-phenanthroline has already been described in the literature, exhibiting dose-dependent response, and its possible mechanism of action is influenced by free radicals, such as ROS, suggesting the oxidative pathway of copper redox cycling as responsible for DNA damage^22,55^. Souza et al. proposed that the ability to cleave DNA through the oxidative pathway is controlled by the type of *N*,*N*-donor linked to the complex^22,52^.

According to Simunkova et al., in oxidative environments such as in the presence of H_2_O_2_ (a condition that stimulates the Fenton reaction), dose dependence was observed in the nuclease activity of the copper (II) complexes and total conversion of the supercoiled plasmid (form I) into linear (form III) and circular (form II) plasmids. Furthermore, as observed in our results, all copper complexes studied by these authors were also able to cleave plasmid DNA, even in the absence of H_2_O_2_. The authors suggested that in addition to redox cycling, copper complexes also induce DNA breaks by other mechanisms, such as intercalation^55^.

We also believe that complex **1** has redox cycling as one of the mechanisms of action responsible for DNA damage and also that the Fenton reaction is also responsible for ROS generation; in this case, the hydroxyl radical attacks the DNA inducing single or double breaks, as shown in the equation below:

$$({\text{Cu}}^{ + } )\left( {4 - {\text{fh}}} \right)\left( {{\text{phen}}} \right) + {\text{H}}_{2} {\text{O}}_{2} \to ({\text{Cu}}^{2 + } )\left( {4 - {\text{fh}}} \right)\left( {{\text{phen}}} \right) + {}^{ \cdot }{\text{OH}} + {\text{OH}}^{ - } \left( {\text{Fenton reaction}} \right)$$

Heinrich et al.^53^ used ascorbate as a reducing agent to inhibit the DNA cleavage capacity of copper(II) associated with phenanthroline, proving that its action is mediated by an oxidative mechanism. Several copper complexes analyzed by Singh et al.^56^ also exhibited chemotherapeutic effects via oxidative, hydrolytic, induction of conformational changes in the DNA molecule and also by the induction of excessive ROS production. According to the authors, these variations in the action mechanisms are due to the flexible redox behavior of copper^53,56^.

Şenel et al. also used hydrogen peroxide as an activator of the copper(II) complexes and observed the same induction of plasmid cleavage, whereas the addition of DMSO as a hydroxyl radical scavenger induced a reduction in the nuclease activity of copper(II) complexes, suggesting the involvement of the hydroxyl radical in the DNA damage pathway. They also emphasize that the presence of aromatic rings, such as those from 1,10-phenanthroline, adequately serve as a partial responsible for the interaction between the complex and the plasmid^54^.

Copper complexes with Schiff bases have shown oxidative damage to DNA and a mechanism based on Fenton-type reaction has also been suggested (ZEHRA; ROISNEL; ARJMAND, 2019). According to Daravath et al. (2017), the schiff-based copper complex exhibited greater efficiency in causing breaks in form I of plasmid pBR322 DNA compared to cobalt(II) and nickel(II) complexes^51,57^.

Our oxidative stress experiments showed that complex **1** induced ROS generation and lipid peroxidation. The oxidative intracellular environment is very harmful and can be directly associated to the DNA damage. In fact, copper is a redox active transition metal and copper complexes are widely known to affect redox cycling^58^.

Chemotherapeutic agents are known for their ability to alter the proliferation of tumor cells and Complex **1** was able to trap Sarcoma 180 cells in G0/G1 phase, demonstrating its antiproliferative potential (Fig. 6). Cells can be in a stationary state (G0) or in a division cycle, which can be divided into four phases (G1, S, G2 and M). Cell cycle is controlled by checkpoints, which ensure cell integrity. If any environmental or molecular problem is detected, the advancement of the cell cycle is blocked^59^. Generally, cells that suffer double- or single-strand breaks in DNA are paralyzed in the G0/G1 or G2 phases^60^. Several copper complexes exhibit the ability to degrade tumor cell DNA^22,61,62^, and such ability was detected in complex **1**. The damage on DNA is believed to be responsible for this cell cycle arrest. Many other copper complexes are also described as having such antiproliferative potential^22,29–31^ trapping cells in G0/G1 and G2.

In addition to the cytostatic capacity, another desirable characteristic for new chemotherapy drugs is the induction of death in a programmed and controlled way, such as apoptosis^32^. In order to evaluate the potential of the apoptosis induction of complex **1**, melanoma B16F10 and sarcoma 180 were treated with the complex and the genes from the apoptotic pathway BCL-2 and Caspase 3 had their expression analyzed with real-time PCR. The downregulation of BCL-2 and the upregulation of Caspase 3 observed in melanoma B16F10 cells indicate that these cells underwent apoptosis after complex **1** treatment. Caspases are initiating and executing proteins of apoptosis. They are present in the cytosol as inactive pro-enzymes, becoming active after proteolytic cleavage. There are two types of caspases, initiators, and effectors, and caspase 3 is considered effector, that is, it results in the apoptotic process^63,64^.

In sarcoma 180, the gene of caspase 3 was also upregulated at the 1.5 µM treatment, but differently from the observed in melanoma B16F10, the expression of BCL-2 was upregulated. An explanation for this event is that in mice, overexpression of the protooncogene BCL-2 attenuates the generation of reactive oxygen species^65^. In the literature, it is known that ROS concentration is intrinsically higher in sarcoma 180 cell line^66^ and we have compared ROS levels between these two cell lines, detecting levels of ROS four times higher in sarcoma 180 than in melanoma B16F10 (See supplementary Fig. S4 online). This high concentration of ROS observed in sarcoma 180 may be related to a more dynamic facility to express BCL-2 to relieve oxidative stress in cases of free radical elevation.

In order to find out whether complex **1** would be effective against a human tumor lineage, we used HeLa cells. Complex **1** proved to be a potent apoptosis inducer in tumor cells, as it was observed in many other copper complexes that exhibit this property^22,29,32,61,62^, which is the safest mechanism of action of a drug, since apoptosis is a controlled event^30^. The genomic DNA degradation event triggered by complex **1** is believed to be the responsible for the induction of apoptosis, as described by other authors^22,31,45^.

One of the strategies to overcome the limitation of already-existing chemotherapy treatments is to explore the activation of ROS-dependent autophagy, which may end up in directing the apoptotic pathway^45^. The complex **1** caused relevant oxidative stress in Sarcoma 180 tumor cells, what motivated us to investigate whether **1** would be able to activate the autophagic pathway in these cells. Morphologically, this type of cell death is marked by massive cytoplasmic vacuolization^67,68^ since, during the process, intracellular and extracellular substrates are delivered to the lysosomes for degradation or recycling^69,70^. Studies demonstrated that autophagy is a mechanism to alleviate cellular stress. However, when overcoming a certain point there is hyperactivation of this system, and apoptotic pathway is activated^45,71,72^. Complex **1** behaved similarly to other copper complexes that induce hyperactivation of the autophagic pathway^22,31,45^. It has been described that almost all classes of anticancer therapy, including agents that cause DNA damage, affect the autophagy mechanism^73^.

**Figure 9:**
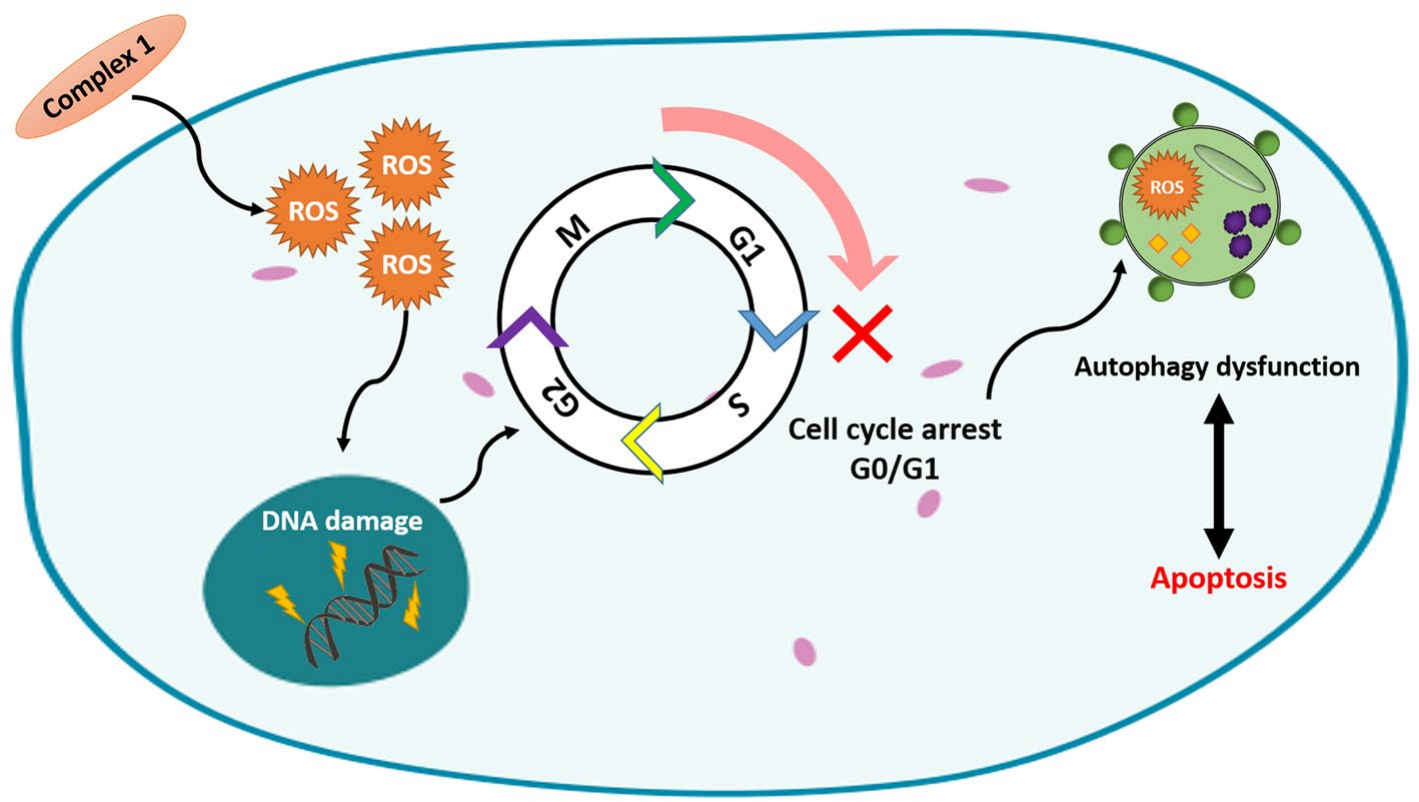
Proposed mechanism of action of complex **1**.

Taken together, we summarized in Fig. 9 a proposed mechanism of action of complex **1**. In summary, we here demonstrated the in vitro antitumor activity of complex **1** highlighting its cytotoxic effect on cancer cell lines. The chemical compound elicited cell cycle arrest, promoted ROS generation, induced genomic DNA damage and triggered cell death through apoptotic and autophagic mechanisms. Complex **1** showed high selectivity in vitro, making it a promising candidate as chemotherapeutic agent. Copper as chemotherapeutic agents has been explored and we suggest that the combination of redox-active molecules, such as complex **1**, with conventional therapy can be useful to improve oncologic treatments and overcome resistance mechanisms. However, additional assays are needed to verify the anti-tumor efficacy of complex **1** in other animal models, to assess its toxicity and mechanism of action.

## Methods

### Starting materials

All chemicals were purchased from Merck and were used as received unless otherwise stated. Doxorubicin hydrochloride (DXR) was used in the present study as a positive control at 0.4 mM (diluted in autoclaved reverse osmosis water) for in vivo tests. DXR at 0.4 mM was previously able to generate ROS and induce homologous recombination in *Drosophila melanogaster* through topoisomerase inhibition^74–77^.

### Preparation of the Cu(II) complexes

The complexes **1** and **2** already described in the literature were prepared according to the published procedure^16^. Elemental analysis (CHN) was used to verify the purity of these complexes.

### Cell lines

Two murine tumor cell lines (Sarcoma 180 and melanoma B16F10), a murine non-tumorigenic cell line (myoblast C2C12) and a human adenocarcinoma (HeLa) were used in this work (purchased from ATCC, reference number CRL-6475). Cell lines were grown in vitro with RPMI-1640 medium, containing 10% fetal calf serum (FCS), 25 mM HEPES, 1% penicillin–streptomycin (10,000 U/mL and 10,000 µg/mL) and 2 mM l-glutamine. Cell lines were kept in a humidified atmosphere of 5% CO_2_ at 37 °C.

### In vitro anti-tumor activity using resazurin assay

Cells were seeded in 96-well plates (1 × 10^5^ cells/well) and incubated with different concentrations of complexes **1** and **2** (ranging from 1 to 1000 µM) or quercetin (ranging from 1 to 2000 µM) for 24 h at 37 °C and 5% CO_2_. Cisplatin was used as positive control (100 µM) and RPMI-1640 medium as negative control. After treatment, cells were incubated with resazurin solution (0.1 mg/mL, 20 µL/well) at 37 °C for 6 h, and the absorbance was later read at 570 nm and 600 nm. All procedures were carried out in triplicate of three independent experiments. Cell viability was determined according to the following equation: viability (%) = [(80,586 × Abs_570_ sample) − (117,216 × Abs_600_ sample)]/[(80,586 × Abs_570_ control) − (117,216 × Abs_600_ control)] × 100^78^. The selectivity index (SI) was obtained using the formula SI = IC_50_ of non-tumorigenic cells/IC_50_ of tumor cells ^24^.

### Analyses of oxidative stress

#### Measurement of intracellular reactive oxygen species (ROS)

The ROS production was based on the intracellular peroxide-dependent oxidation of 2′,7′-dichlorodihydrofluorescein diacetate (H_2_DCF-DA) (Invitrogen catalog number: D399) to form the fluorescent compound 2′,7′-dichlorofluorescein (DCF), as previously described^79^. Briefly, sarcoma 180 cells (1 × 10^6^ cells/mL) were seeded in a 12-well microplate. The tumorigenic cells were treated with different concentrations of complex **1**, cisplatin (40 μM) and quercetin (62.5 μM). Experiments were conducted for 24 h in a CO_2_ incubator at 37 °C. Hydrogen peroxide (H_2_O_2_) was used as a positive control of ROS production. After treatment, cells were harvested and washed with PBS and, under darkness, the cells were incubated in 100 μL of PBS containing the probe (20 μM) for 45 min at 37 °C. Cells that did not receive the treatments with the complex **1** and cells that did not receive the (DCFH-DA) probe were considered as negative controls. Cells were analyzed with a Cytoflex flow cytometer (Backman Coulter, United States) and the results were obtained by using CytExpert Software for the Cytoflex Platform. The data were expressed as percentage of labeled cells.

#### Lipid peroxidation analyses

Sarcoma 180 cells (2 × 10^7^ cells/mL) were treated with complex **1** in three concentrations (1 µM, 4 µM and 20 µM) for 24 h. Lipid peroxidation was assessed by the thiobarbituric acid-reactive substances (TBARS) assay described by Buege and Aust^80^. After the incubation time with the treatment, the cells were homogenized in ice-cold PBS, and lysed through three cycles of freezing and thawing. The homogenate was mixed with TCA, trichloroacetic acid (28%w/v in HCl 0.25 M), TBA, thiobarbituric acid (1% in acetic acid 0.25 N) and BHT, Butylhydroxytoluene (125 mM in ethanol), heated for 15 min at 95 °C and then placed in an ice bath.

Subsequently, the precipitate was removed by centrifugation at 10,000*g* for 15 min at 4 °C, and the absorbance of the supernatant determined at 535 nm in a spectrophotometer. TBARS levels were calculated using 1,1,3,3-tetramethoxypropane as the standard for constructing the calibration curve. Total protein concentration in the samples was determined according to Lowry et al.^81^. Results were expressed as nmol of TBARS/mg protein.

### DNA cleavage experiments

#### Genomic DNA fragmentation assay

Sarcoma 180 tumor cells (1 × 10^6^ cells/mL) were seeded in 12-well culture plate with 250 µL of medium RPMI-1640 (10% v/v of FCS) and were treated for 24 h with complex** 1** at 1 µM, 4 µM and 20 µM. The genomic DNA (gDNA) was extracted using the universal Quick-DNATM kit (Zymo research-USA), and quantified in NanoDrop (Thermo Fisher Scientific). In order to verify gDNA degradation, 50 ng of gDNA extracted from each treatment was analyzed in an agarose gel (0.8%) stained with ethidium bromide. The electrophoresis was carried out for 2 h at 80 V and the DNA bands were visualized using a UV transilluminator Image Quant 150 (GE Healthcare) and photographed.

#### Plasmid DNA cleavage assay

The capacity of complex **1** cleave plasmid DNA was checked by agarose gel electrophoresis. The plasmid siSTRIKE U6 Hairpin Cloning System (Human)—hMGFP (PROMEGA, USA) was cloned and expanded in the *E. coli* DH5α eletrocompetent bacteria and extracted using the kit PureLink HiPure Plasmid Midiprep Kit. A total amount of 30 ng/µL of plasmid was treated with complex **1** at concentrations of 5 µM and 20 µM in PBS 1× containing or not dimethyl sulfoxide (DMSO—0.05%) and hydrogen peroxide (H_2_O_2_—15 mM). Plasmids without treatment with complex **1** were considered as negative control, the treatment with the restriction enzyme *Nhe* I was considered as positive control for double-strand breaks and treatment with H_2_O_2_ (15 mM) was performed as a control for oxidative environment. The reaction mixtures were incubated at 37 °C for 12 h and then 5 µL of loading buffer (0.25% bromophenol blue, 0.25% xylene cyanol, 30% glycerol, 10 mM EDTA) was added. The samples were subjected to electrophoresis on a 0.8% agarose gel containing 0.05% ethidium bromide (10 µg/mL) in 90 mM Tris–borate buffer, pH 8.0, 20 mM EDTA (0.5× TBE). The gel was subjected to 80 V for 3 h and photographed under ultraviolet light. The bands were quantified using ImageJ software version 1.53 k (Java 1.8.0_172) in order to identify the percentage of DNA in each band of the gel column.

### Analyses of cell cycle progression

Sarcoma 180 tumor cells (1 × 10^6^ cells/mL) were seeded in a sterile 12-well plate and treated for 48 h with three concentrations of complex **1** (1 µM, 4 µM, 8 µM). Untreated cells were considered as negative control. The cells were centrifuged for 5 min at 2000 rpm. The pellet was washed in PBS 1× (repeated twice), fixed in ethanol 70% (ethanol/PBS), and stored at 4 °C overnight. The cells were then centrifuged for 5 min at 2000 rpm and the pellet was resuspended in PBS containing 10 µg/mL of PI and 100 µg/mL of RNase. The cells were incubated in the dark for 45 min at 37 °C. After the incubation time, the cells were taken for analysis with the BD Accuri C6 cytometer on the FL2 channel. The data resulting from the cytometry were analyzed using the FloJo software (version 10).

### Reverse transcription and qPCR

After treatments, the total RNA of each sample was extracted using the TRIzol Reagent (Life Technologies), following the manufacturer's recommendations. Quantification was performed in a NanoDrop 1000 spectrophotometer (Thermo Fisher Scientific) and the 260 nm/280 nm ratio was also used as a qualitative parameter. Then, total RNA was stored at − 80 °C for subsequent analyses. For reverse transcription, 2 μg of total RNA was denatured at 70 °C for 5 min, cooled on ice for 5 min and added to a mix containing 10 μL of GoScript Reverse Transcription Oligo(dT) (A2791, Promega), as recommended by the supplier. Control reactions were also prepared to check for possible exogenous contaminants. The samples were incubated in an Arktik thermocycler (Thermo Fisher Scientific) and the conditions used were 25 °C for 5 min, 42 °C for 60 min and 70 °C for 15 min. cDNA was stored at -20 °C for further amplification.

The transcripts of BCL2 apoptosis regulator (BCL-2), Caspase 3 (CASP3), Autophagy-related 5 (ATG5) and Autophagy-related 7 (ATG7) genes were quantified by qPCR, using the Glyceraldehyde-3-phosphate dehydrogenase (GAPDH) gene as reference (Table 2). For each target, a relative standard curve was constructed to validate the comparative Cq method. Reactions were prepared to a final volume of 10 μL containing cDNA, primers and GoTaq qPCR Master Mix (A6001, Promega), and carried out in the 7300 Real Time PCR System (Applied Biosystems) equipment. Cycling conditions were denaturation of 95 °C for 1 min followed by 40 cycles with denaturation at 95 °C for 15 s and annealing/amplification a 60 °C for 1 min.

**Table 2:** Sequences of primers used for amplification of BCL2 apoptosis regulator (*BCL-2*), Caspase 3 (*CASP3*), Autophagy-related 5 (*ATG5*), Autophagy-related 7 (*ATG7*) and Glyceraldehyde-3-phosphate dehydrogenase (*GAPDH*) genes.

Genes	Sequences 5′–3′
*BCL-2*	F: CGCCGGGCTGGGGATGACTTCT R: CACTTGTGGCCCAGGTATGC
*CASP3*	F: AGACCATACATGGGAGCAAGTC R: CAGAGCGAGATGACATTCCAG
*ATG5*	F: GAAGAATGACAGATGACAAAGATGTGC R: GCTGACTCTTGGCAAAAGCAAATA
*ATG7*	F: GATGTATGGACCCCAAAAGGCT R: CCAGCCCATCAGTGTCCTAG
*GAPDH*	F: GAAGGTCGGTGTGAACGGATT R: TGCCGTGAGTGGAGTCATACTG

### Apoptosis assay

HeLa cell line (1 × 10^5^ cells/well) was seeded in 96 well sterile microplate—and treated with three concentrations (1 µM, 4 µM and 10 µM) of complex **1** for 48 h. After the treatment time, the cells were resuspended in a Hoechst 33,342 solution (10 µg/mL) and PI (2.5 µg/mL), incubated in the dark at 37 °C for 10 min. Using an EVOS fluorescence microscope (Thermo Fisher Scientific, United States), at least 300 cells were counted for each replicate (totaling 900 cells per treatment). The cells were evaluated according to their chromatin structure and staining. The percentages of early apoptosis, late apoptosis and necrosis were determined from the total number of counted cells as previously described^82,83^.

### Autophagy assay

In a sterile 24-well culture plate, the Sarcoma 180 tumor cells (2 × 10^5^ cells/well) were treated with complex **1** in three concentrations (1 µM, 4 µM and 20 µM), hydrogen peroxide 1 mmol/L (positive control) or culture medium (negative control). The cells were treated for 24 h and afterwards Monodansylcadaverine (MDC)—Sigma-Brazil—(0.05 mM/well) was added and incubated at 37 °C, CO_2_ 5% for 60 min in the darkness. The samples were collected, centrifuged and the pellet washed three times with ice-cold PBS 1×. The pellet was resuspended in a solution of PBS + Glycine (0.1 M) and 500 µL of this solution of cells-PBS-Glycine was placed in a 24-well plate and immediately analyzed under a fluorescence microscope (Zeiss L5M510, Germany). Four microscopic fields were randomly analyzed at 200× magnification and fluorescence intensity was captured by Image J software.

### Statistical analysis

The IC_50_ or Half Maximal Inhibitory Concentration (concentration that inhibits 50% of cell growth) was determined using the program GraphPad Prism 6.0 (GraphPad Software Inc., La Jolla, California, USA), from a non-linear regression, where the percentage of cell viability was determined as a logarithmic function of tested concentrations, assuming a 95% confidence interval (p < 0.05). The statistical analyses of cell cycle progression and autophagy assays were performed with one-way ANOVA with Bonferroni’s multiple comparisons post-test. Statistical analyses for ROS and apoptosis assays were performed with two-way ANOVA with Bonferroni’s multiple comparisons post-test. The lipidic peroxidation analysis was performed by T-test. Data were considered statistically significant when p < 0.05.

## Supplementary Information

Supplementary Figures.
