# Supplementary material for: A selective CuII complex with 4-fluorophenoxyacetic acid hydrazide and phenanthroline displays DNA-cleaving and pro-apoptotic properties in cancer cells
Source: Sci Rep. 2021 Dec 27;11:24450. doi: 10.1038/s41598-021-03909-1 (PMC8712526; doi:10.1038/s41598-021-03909-1)

**A selective copper^II^ complex with 4- fluorophenoxyacetic acid hydrazide and phenanthroline displays DNA-cleaving and pro-apoptotic properties in cancer cells.**

Pedro Henrique Alves Machado^a^, Drielly Aparecida Paixão^b^, Ricardo Campos Lino^a^, Tiago Rodrigues de Souza^a^, Nayara Júnia de Souza Bontempo^a^, Luana M. Sousa^b^, Fernanda Van Petten de Vasconcelos Azevedo^a^, Priscila Capelari Orsolin^d^, Paula Marynella Alves Pereira Lima^a^, Isabella Castro Martins^a^, Joyce Ferreira da Costa Guerra^a^, Samuel Cota Teixeira^c,*^, Thaise Gonçalves Araújo^a^, Luiz Ricardo Goulart^a^**^♱^**, Sandra Morelli^a^, Wendell Guerra^b^, Robson J. de Oliveira Júnior^a,*^

^a^Instituto de Biotecnologia, Universidade Federal de Uberlândia, Uberlândia - MG, Brasil

^b^Instituto de Química, Universidade Federal de Uberlândia, Uberlândia - MG, Brasil

^c^Departamento de Imunologia, Instituto de Ciências Biomédicas, Universidade Federal de Uberlândia, Uberlândia - MG, Brasil

^d^Centro Universitário de Patos de Minas – UNIPAM, Patos de Minas-MG, Brasil

**^♱^**Deceased October 24, 2021

*Corresponding authors:

Samuel Cota Teixeira, Ph.D and Robson J. de Oliveira Júnior, Ph.D

Departamento de Imunologia

Instituto de Ciências Biomédicas,

Universidade Federal de Uberlândia

Campus Umuarama, Av. Para, 1720 | 38400239

Uberlândia-MG, Brazil

Phone: +55 34 32258569

E-mail addresses: samuel.teixeira@ufu.br and oliveirajunior@ufu.br

**Supplementary Figure S1 online.** Sigmoidal dose-response curves of treatment with quercetin in melanoma B16F10 cell line (red points) and sarcoma 180 (black points). Each point represents the means ± standard deviation for n = 4 replicates.





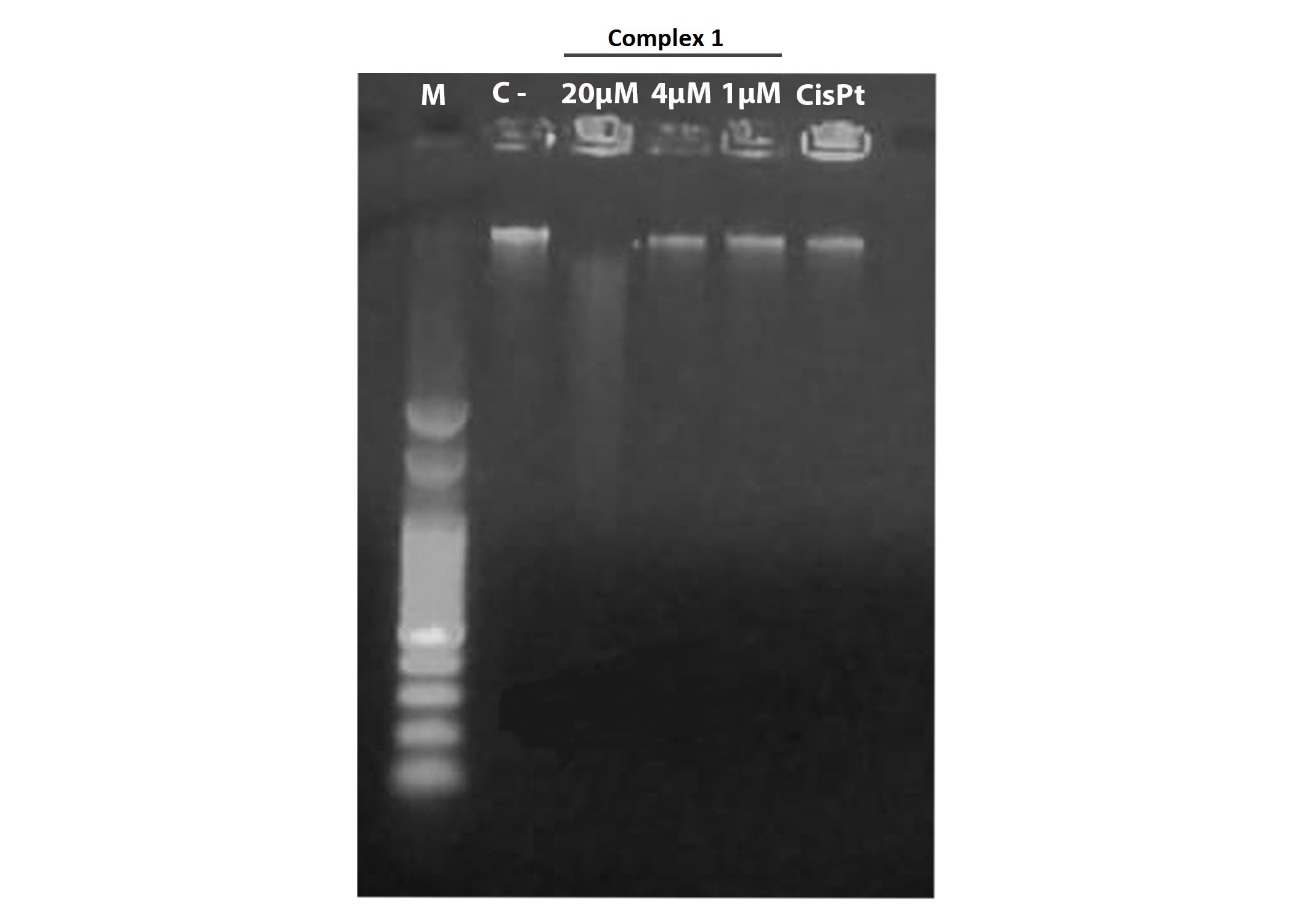
**Supplementary Figure S2 online.** Electrophoresis in agarose gel demonstrating the capacity of complex **1** to induce the degradation of genomic DNA in Sarcoma 180 tumor cells at the concentration of 20 μM. M = 100 base pair molecular marker; C- = Medium RPMI-1640; CisPt = 40 μM cisplatin. **Full-length gels are presented in Supplementary Figure S5.**

**Supplementary Figure S3 online**. A) Analysis of cell death of HeLa cell line treated with complex **1** (1 μM, 4 μM and 10 μM). Data represent the means ± the standard error. * p <0.05. ** p <0.01, *** p <0.001 and **** p <0.0001 (compared to the negative control by two-way ANOVA followed by Bonferroni´s post-test). B) Time lapse of the apoptotic process of the HeLa cell line treated with 100 μM of Complex **1**.


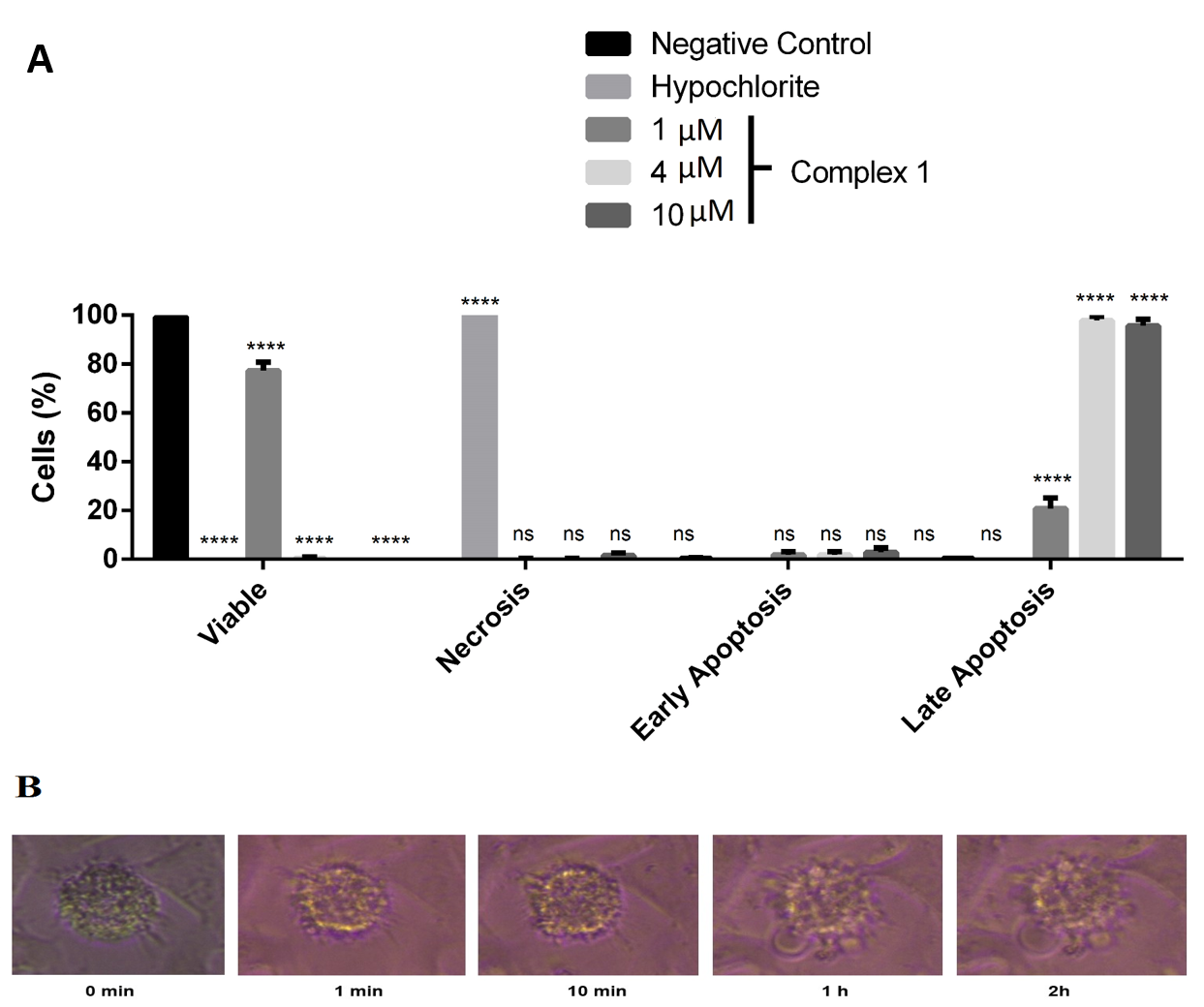


**Supplementary Figure S4**. Comparison between ROS levels of melanoma B16F10 and sarcoma 180. The columns represent the median fluorescence intensity of H2DCFDA. Data represent the means ± the standard error. **** p <0.0001 (groups compared through T-test).





**Supplementary Figure S5.** A) Full-length gel photo of S2 figure; B) Full-length gel photo of 6B figure


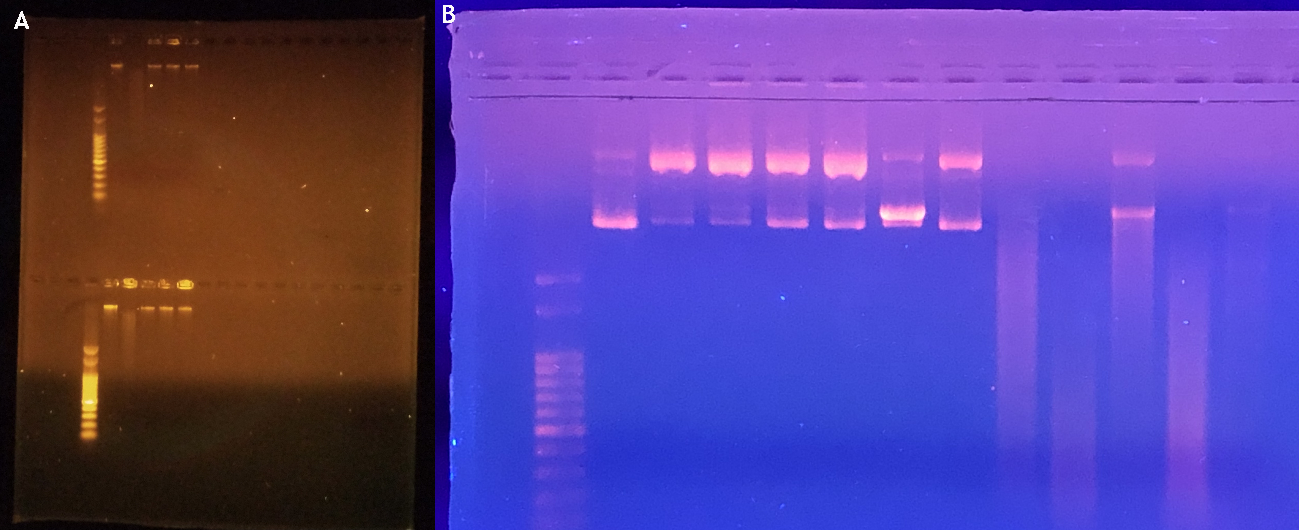

Supplement: Supplementary file 1 — Supplementary Figures. [file 41598_2021_3909_MOESM1_ESM.docx]
